# 支气管镜介入技术在外周肺病变中的应用

**DOI:** 10.3779/j.issn.1009-3419.2016.08.13

**Published:** 2016-08-20

**Authors:** 慧 王, 礼年 黄

**Affiliations:** 233000 蚌埠, 蚌埠医学院第一附属医院呼吸与危重症医学科 Department of Respiratory and Critical Care Medicine, the First Affiliated Hospital of Bengbu Medical College, Bengbu 233000, China

**Keywords:** 支气管镜, 外周肺病变, 超细支气管镜, 支气管内超声, 导航支气管镜, 荧光共聚焦显微镜, Bronchoscope, Peripheral pulmonary lesions, Ultrathin bronchoscopy, Endobronchial ultrasonography, Navigation bronchoscopy, Fibered confocal fluorescence microscopy

## Abstract

肺癌是全球癌症相关死亡的主要原因, 肺癌的治愈率很低, 不仅因其自身攻击性, 还因对肺癌筛查的忽视。随着肺部筛查手段的不断进展, 肺外周病变的检出率逐渐提高, 当前对外周肺病变进行诊断的最常用方法是经支气管行支气管镜检查或计算机断层扫描(computed tomography, CT)引导下经皮穿刺针吸/活检, 然而对于外周肺病灶, 支气管镜检查有较低的诊断率, 经皮穿刺检查有较高的气胸发生率, 因此, 使用安全、微创的方法对外周肺病变进行组织确诊是临床工作者将面临的挑战。新型支气管镜介入诊断技术已逐渐用于临床, 这些技术可有效提高外周肺病变的诊断率, 缩短诊断时间, 使患者获得及时有效的治疗。本文将现有的技术进行简要综述以帮助临床医生尝试应用这些微创技术。

## Abstract (en)

Lung cancer is the leading cause of cancer-related mortality worldwide.A low cure rate of lung cancer is not only attributed to intrinsic aggressive biological behavior, but also little attention to lung cancer screening.With lung screening methods continuous progress, peripheral pulmonary lesions detection rate gradually increased.Currently, a transbronchial approach using a bronchoscope or computed tompgraphy (CT) guided transthoracic needle aspiration/biopsy have been the most generally accepted methods for diagnosing peripheral pulmonary lesions.However, conventional bronchoscopy has a poor diagnostic yield and CT-guided approach has high rates of pneumothorax for such peripheral pulmonary lesions.Therefore, clinicians will be challenged with the task of providing the means to provide a safe and minimally invasive method of obtaining accurate tissue diagnostics for the pulmonary peripheral lesions.New bronchoscopic interventional diagnosis technologies have recommended in clinical gradually.They can effectively improve the peripheral pulmonary lesions diagnosis rate, shorten the time of diagnosis, and make the patients get timely and effective treatment.In this paper, we reviewed briefly available technologies to aid clinicians in attempts at minimally invasive techniques.

外周肺病变(peripheral pulmonary lesion, PPL)是指位于中外2/3肺野的外周肺病灶。最近美国国家癌症研究所进行的一项全国肺癌筛查试验指出:对肺癌高危患者行低剂量计算机X射线断层扫描(computed tomography, CT)筛查有利于降低肺癌患者的死亡率^[1]^, 这预示着PPL的筛检率将不断提高。以往PPL的诊断依靠常规支气管镜下活检、针吸、刷检、灌洗, 经皮穿刺活检、针吸, 手术切取病灶活检等。然而, 支气管镜对PPL的诊断受病灶大小的影响, 对 > 20 mm和 < 20 mm的周围型肺癌诊断灵敏度分别为63%和34%^[2]^; 经皮穿刺检查对周围型肺癌诊断灵敏度为90%, 但有15%的气胸发生率, 7%需肋间引流, 且增加了患者的辐射暴露^[2]^; 胸腔镜下或开胸手术病灶活检虽为金标准, 但创伤较大且花费高; 若为恶性病变, 一味的观察等待可能会延误治疗, 我国最近统计的肺癌发病率为35.23/10万, 死亡率为27.93/10万, 肺癌病死率较高, 故提倡早期诊疗^[3]^。

随着内镜技术的不断更新, 新型支气管镜介入诊断技术有望解决这一问题, 它能够更早期地对PPL进行定位、定性诊断, 有利于患者及时治疗、改善预后。本文将对各项技术的作用原理、应用利弊及各技术间联合应用等相关问题进行综述。

## 1 超细支气管镜

传统支气管镜的外径大约4.5 mm-6.3 mm, 只能访问到4级-5级支气管, 远端支气管处的病变无法直视, 仅可盲检、盲刷, 超细支气管镜(ultrathin bronchoscopy, UTB)在一定程度上解决了这些问题。外径 < 3 mm的UTB于20世纪80年代问世, 最初因内置通道的缺乏仅用于观察外周气道, 后来研究设计了内置通道, 完善了刷检、灌洗及活检等功能。现在采用的UTB先端部外径多在2.8 mm-3.0 mm, 可直视至8级-10级支气管, 有报道称最远至12级支气管^[4]^, 并可对上叶尖、后段, 下叶背段的病灶进行检查, 其工作通道直径1.2 mm-1.7 mm, 允许1.5 mm的活检钳、1.4 mm的超声探头、1.0 mm的毛刷进入通过, 它上偏角度180°, 下偏130°, 视野范围为90°-120°, 可视距离约2 mm-50 mm, 可用于部分无法经FB探及的PPL。

Yamamoto等^[5]^进行了一项研究, 对32例经常规支气管镜检查下快速细胞学结果为阴性的PPL患者先行活检, 再行UTB检查, 并做UTB下快速细胞学检查及活检, 最终共确诊22例, 诊断率为68.8%, 22例中有9例由常规支气管镜活检确诊、19例可经UTB检查确诊, 有13例(13/22, 40.6%)仅由进一步UTB检查获得诊断, 此研究证明, 对PPL的诊断UTB比常规支气管镜更有优势, 可作为支气管镜的有利补充。Oki等^[4]^对310例患者进行了随机对照试验, 比较了UTB(外径3.0 mm, 内径1.7 mm)和带引导鞘的细支气管镜(thin bronchoscope with a guide sheath, TB-GS)(外径4.0 mm, 内径2.0 mm)对 < 30 mm的PPL诊断率, 结果显示:UTB的诊断率为74%(111/150), TB-GS的诊断率为59%(92/155), 差异有统计学意义(*P*=0.044);且UTB比TB-GS能到达更远端支气管, 对外1/3肺野的病灶有更高诊断率(*P*=0.002)。

UTB检查时患者的耐受性好, 较易配合, 其外径纤细, 可直视下对PPL进行活检, 既提高了诊断率, 又可避免患者经皮穿刺导致气胸的风险^[4, 6]^。但UTB的应用也有其局限性:UTB外径纤细、灵活性较高, 若固定不稳或患者稍咳嗽, 可致管镜偏移, 增加了UTB在外周肺野的操控难度; 可通过狭小工作通道的取样工具有限, 1.7 mm内径的UTB可与支气管内超声(endobronchial ultrasonography, EBUS)联合应用, 但1.2 mm内径的UTB尚无与之相适的EBUS设备, 由于活检钳较小, 钳取组织量少且常有脱落, 使得活检往往差强人意; UTB内径狭小, 吸引较差, 少量出血及分泌物也可导致视频图像模糊^[7]^。因此, UTB并非PPL的首选检查, 当常规支气管镜无法探及病灶时, UTB可作为内镜检查的有力补充。

## 2 支气管内超声

带引导鞘的支气管内超声(endobronchial ultrasonography with a guide sheath, EBUS-GS)在2004年最先报道用于PPL的诊断, 径向EBUS结合一个引导鞘, 经支气管镜的工作通道到达病灶周围, 通过一个20 MHz的微型超声探头, 产生肺实质周围360°的超声图像, 确定靶区后于此位置固定导鞘, 并将其作为一个延长的工作通道, 在退出探头后, 将活检设备置入引导鞘, 在靶区重复取样。取样后引导鞘内可残留少许样本, 有研究指出, 对残存样本冲洗后行细胞学检查, 比支气管灌洗和刷检有更高诊断率^[8]^。EBUS-GS不仅可对病灶进行定位, 还可初步评估病变性质, Kurimoto等^[9]^对EBUS图像下PPL的内部结构进行研究指出, 图像为均质型表现的病灶92.0%为良性, 表现为点线状高回声或异构型的病灶99.0%为恶性。另外, EBUS探头空间分辨率 < 1 mm, 探测深度为4 cm-5 cm, 可显示支气管壁的层次, 对病变浸润深度进行评估, 提高了PPL的诊断率。

Wang等^[10]^对39项有关支气管镜诊断技术的研究进行汇总, 指出EBUS-GS检查对肺结节有最高的汇集诊断率(73.2%)。Steinfort等^[11]^进行了一项1, 420例的荟萃分析, 指出EBUS对周围型肺癌的诊断特异性为100%, 灵敏度为73%。Kurimoto等^[12]^对150例行EBUS-GS的PPL患者进行研究, 得出77%的诊断率, 进一步分析指出EBUS-GS对≤20 mm的PPL也有较高诊断率(58/81, 71.6%), 探头位于病灶内比位于病灶旁的诊断率明显提高(*P* < 0.000, 1)。Tay等^[13]^研究了EBUS诊断PPL的影响因素, 指出病灶大小、良恶性、距肺门的距离影响病灶的可视率, EBUS探头下可视病灶的诊断率为65%(101/155), 不可视病灶诊断率为20%(8/41), 差异有统计学意义(*P*=0.000, 1)。Guvenc等^[14]^对760例进行EBUS检查的患者CT图像进行分析, 指出CT上存在支气管充气征时, EBUS检查往往可伸入该充气支气管, 使探头更接近病灶, 从而提高了EBUS图像的可视率, 支气管征阳性组病变的诊断率为72%(424/618), 阴性组诊断率为20%(29/142), 差异有统计学意义(*P* < 0.001), Minezawa和Evison等的二项研究也指出, CT上有支气管征象是EBUS引导支气管镜检查成功与否的重要预测因子^[15, 16]^。因此, EBUS-GS程序有助于提高PPL的诊断率, 且对较小病灶受益更大, 探头位置及有无支气管征是影响诊断率的重要因素。2013年美国胸科医师协会(American College of Chest Physicians, ACCP)提出, EBUS提高了纤支镜对外周肺病变的诊断能力, 且不增加操作风险, 推荐在侵入性操作前先行EBUS检查, 对病灶进行定位与评估^[2]^。

EBUS图像对气管旁的血管影也有较好的显示, 活检定位时可尽量避开血管, 活检后引导鞘在病灶处固定2分钟, 可减少出血的风险^[12]^, 2010年日本呼吸内镜协会进行了一项全国范围的调查^[17]^, 指出支气管镜对孤立性PPL检查时并发症发生率为1.55%(937/60, 275)(依次为出血0.63%, 气胸0.44%, 感染0.25%), 而2015年日本东京国家癌症中心的统计指出^[18]^, EBUS-GS检查并发症发生率为1.3%(气胸0.8%, 感染0.5%, 无明显出血), 说明EBUS-GS的应用较其他程序明显降低出血发生风险。另有学者将EBUS-GS检查与CT引导经皮肺穿刺进行比较, 指出EBUS检查的诊断准确性不明显低于CT引导经皮穿刺(87.5% *vs* 93.3%, *P*=1.0), 但其并发症发生率却明显低于后者(3% *vs* 27%, *P*=0.03), 且减少了检查时的辐射暴露^[19]^。

## 3 导航支气管镜

因支气管树分支繁多, 在有限的时间内很难保证支气管镜能到达外周肺病变部位进行检查, 依赖CT数据直观地选择支气管路径往往是不准确的, 为了克服这个问题, 导航支气管镜检查应用于临床, 目前使用的有虚拟支气管镜导航(virtual bronchoscopic navigation, VBN)和电磁导航支气管镜(electromagnetic navigation bronchoscopy, ENB)。

### 3.1 虚拟支气管镜导航

是依据仿真支气管镜的原理设计, 通过计算机软件将二维螺旋CT的数据编写成虚拟支气管(virtual bronchus, VB)图像, 在确定PPL位置后自动生成一条通往病灶的支气管路径, 由于VB图像与真实图像极为相似, 支气管镜可通过此路径到达目标病灶。VB图像下支气管的解剖细节取决于获取的CT数据, CT层厚和卷积函数的阈值设定影响VB图像的质量, 阈值选择不当, 将不能准确区分气道壁和管腔, 导致支气管分支的缺失。新近开发的VBN系统(Bf-NAVI; KGT, Olympus Medical Systems, Tokyo, Japan)可自动调整合适的阈值, 完成VB图像的形成及路径的选取, 显示至4级-12级支气管, 并可自动调整VB图像, 使其与真实支气管图像吻合。

Eberhardt等^[20]^对25例PPL患者进行研究指出, 所有病例均可由VBN引导支气管镜到达病灶处活检, 诊断率达80%, 有14例可沿导航路径一次到达病灶处, 明显缩短了检查时间, 仅1例活检后出现少量气胸。对外周病变的检查, 往往需要VBN引导UTB才能到达目标病灶, Asano等^[6]^报道了一项多中心随机对照试验, 评估VBN辅助UTB对PPL的诊断价值, 结果显示VBN辅助组总诊断率为67.1%(112/167), 非VBN辅助组为59.9%(100/167), 差异无统计学意义(*P*=0.173), 但亚组分析显示, 对X线片上不可见病灶、更远端支气管处病灶(右上叶或外1/3肺野), VBN辅助组的诊断率明显高于非VBN辅助, 且仪器到达目标病灶的时间明显缩短, 减少了长时间检查给患者增加的痛苦。

VBN检查类似于常规支气管镜检查, 除了需要特定的计算机软件外, 不需要特定的设备及专业人员的训练, 不明显增加检查成本, 该程序并发症发生率与常规支气管镜检查相当, 没有与VBN直接相关的并发症报道。但是VBN程序也有其局限性, VB图像在检查前获取, 不能对支气管镜进行实时引导, VBN本身不能证实支气管镜是否到达病灶, 需结合透视、CT或EBUS来证实。

### 3.2 电磁导航支气管镜

该设备由四部分组成:电磁板、传感器探头、软件及显示器。ENB也是根据仿真支气管镜原理设计, 操作前通过获取CT数据形成VB图像, 并在VB图像、支气管树及病灶中心做标记, 然后让患者躺于电磁板上, 胸前贴3个传感器, 再将传感器探头通过导管经支气管镜通道置入患者支气管腔内进行校准, 由软件自动生成到达目标病灶的导航线路, 然后通过调节导管使远端探头做360°运动, 沿导航路径行进, 传感器探头有实时定位功能, 引导支气管镜到达目标病灶后固定导管, 退出导丝与探头后对病灶进行取样。ENB比VBN增加了实时定位功能, 但也增加了操作难度及高额耗材。

ENB在欧美国家日益盛行, 我国仍处于起步阶段。2013年ACCP的肺癌诊断指南中提出, ENB对周围型肺癌有71%的诊断率, 有助于提高支气管镜对外周肺疾病的诊断率^[2]^。Gex等^[21]^对15项试验1, 033个行ENB检查的PPL进行荟萃分析, 指出ENB对其总体灵敏度为64.9%, 准确性为73.9%, 对肺部恶性疾病的灵敏度为71.1%, 阴性预测值为52.1%, 此结果与Wang等^[10]^得出的ENB有67%汇集诊断率相近。

ENB程序对PPL的定位率有明显提高, 但诊断率没有达到预期, 考虑与活检设备、图像间误差及医师经验有关。ENB的配套活检设备长期未更新, 活检时往往取样不足。Chen等^[22]^对85个肺部结节的运动进行量化, 指出结节位置在充分吸气末及平静呼气末的平均移动范围约17.6 mm, 位于下叶的结节比上叶的移动更明显, 所以在充分吸气末摄片形成的病灶图像位置不能完全反应支气管镜检查时的实际位置, 这一误差将对ENB的诊断率造成影响。另有研究指出^[23]^, 在虚拟图像标记与探头位置进行校准时, 会产生平均基准目标配准误差, 低的配准误差联合医师的快速现场评价对PPL有较高的诊断率。Lamprecht等^[24]^对112例孤立性肺病灶进行分析也指出, 医师的快速现场评价经验与提高ENB的诊断率有关。Luo等^[25]^最近研究了一种无标记的配准方法, 将导航路径的中心线与传感器探头相关联, 完成电磁跟踪器与虚拟图像的实时配准, 形成动态的虚拟支气管图像, 可明显缩小配准误差, 将有利于提高ENB的诊断率, 具体成效需结合临床进一步评价。

## 4 荧光共聚焦显微镜

荧光共聚焦显微镜(fibered confocal fluorescence microscopy, FCFM)也称“肺泡镜”, 是根据共聚焦显微镜成像原理, 将可弯曲的光纤探头通过支气管镜的工作孔道深入到远端的肺泡管, 利用488 nm波长的激光, 激发组织的荧光特性, 对支气管粘膜结构进行扫描, 即“光学活检”, 其探测范围可深达支气管壁下50 μm, 图像直径约为600 μm, 可探测出支气管壁癌前病变时存在的基底膜网状板纤维结构的变化, 以协助肺部病变的早期诊断。由于FCFM检查范围很小, 不可能检查全部支气管壁, 因此需要先行自荧光纤维支气管镜(automatic fluorescence bronchoscopy, AFB)检查, 对可疑病变部位有选择地进行FCFM检查, 可避免对病变阴性部位活检和重复操作, 在组织损伤最少的条件下, 观察到与癌前病变有关的支气管基底膜变化, 提高支气管镜活检的阳性率。

Thiberville等^[26]^对29名肺癌高危人群先进行AFB检查, 再使用直径1.4 mm的光纤探头对71个可疑病变部位行FCMC检查, 而后取活组织行组织病理学检查, 发现了22个支气管粘膜化生及不典型增生(其中19个发生自体荧光显微结构的改变)、5例原位癌、2例浸润性病变, 发现的27个浸润前病变中有9个发生纤维网状结构的破坏。Rakotomamonjy等^[27]^对支气管粘膜的FCFM图像进行分析指出, FCFM对肺癌有较好的检测率, 未来有望通过FCFM图像正确识别支气管癌前病变的不同级别, 如上皮化生、不典型增生和原位癌。该检查目前仍处于实践阶段, 其准确性及实用性尚有待研究。

## 5 新技术的联合应用

UTB能进入更远端小气道直视病灶; EBUS-GS对病灶进行准确定位与评估; VBN提供虚拟图像引导支气管镜到达病灶; ENB实时引导支气管镜到达目标病灶; FCFM可在微观水平发现早期病变, 各技术间联合可取长补短, 共同提高PPL的诊断率。有学者研究了EBUS-GS引导下经支气管镜针吸活检(transbronchial needle aspiration, TBNA), 应用外带金属导鞘、针长13 mm的活检针抽吸取样, 可明显提高PPL的诊断率, 并可协助恶性病变分期, 且不增加并发症风险, 当探头定位不在病灶中央时, TBNA对提高诊断率更有帮助^[28, 29]^。另有学者^[30, 31]^研究ENB引导TBNA取样, 指出TBNA对肺部病灶有较高诊断价值, 尤其是对较小的病灶, 其准确性往往优于活检。Ost等^[32]^分析了ACCP有关支气管镜诊断PPL的记录数据, 也指出TBNA取样有助于提高PPL诊断率, 其诊断效用优于经支气管镜活检。

Tamiya等^[33]^对68例VBN引导EBUS-GS检查进行研究, 得出77.9%的PPL诊断率, 并提出应用导航技术精确引导后, ≤20 mm和20 mm-30 mm的PPL诊断率分别为74.1%(20/27)、80.5%(33/41), 病灶的大小对其诊断率的影响无统计学意义(*P*=0.534)。Ishida等^[34]^进行一项199例的随机对照研究, 评估VBN引导EBUS-GS对PPL的诊断价值, 指出VBN引导EBUS-GS较单用EBUS-GS的诊断率明显提高(80.4% *vs* 67%, *P*=0.032), 且缩短了检查时间(*P*=0.016)。以上两项研究均用外径4.0 mm的细支气管镜进行检查, 上文Oki等^[4]^阐述了UTB较TB-GS对PPL有更高诊断率, Asano等^[6]^也阐述了对X线片不可见病灶或更远端支气管病灶, VBN引导UTB检查显示了更高的诊断率, 因此, 若将VBN/UTB/EBUS结合, 有望能进一步提高PPL的诊断率。

Eberhardt等^[35]^对118例PPL患者进行随机对照试验, 指出ENB联合EBUS对PPL有88%(35/40)的诊断率, 高于单用EBUS(27/39, 69%)或单用ENB(23/39, 59%)(*P*=0.02), 且不增加并发症的风险。但ENB检查操作复杂、花费较高, Chee等^[36]^设计了一项试验, 对60例PPL患者先使用EBUS-GS定位病灶, 定位失败时再使用ENB辅助EBUS定位, 75%的PPL可经EBUS-GS定位活检, 有15例需要额外行ENB引导, 最终总体定位率可达93%。Steinfort等^[37]^也设计了类似试验, 先对245个PPL病灶行EBUS联合VBN检查, 77%的病灶可准确定位, 对57个未经EBUS定位的病灶额外行ENB检查有17例可获得准确定位, 提高定位率至85%。

## 6 总结

前面我们对用于PPL诊断的最新支气管镜介入技术及各项技术间联合应用进行综述, VBN/UTB/EBUS结合, 是较为推荐的诊断程序, ENB可在VBN、EBUS-GS检查不能定位病灶时考虑使用, 未来FCFM的发展有望在微观水平早期诊断肺部病变。但临床中患者及病灶的个体差异普遍存在, 已有的推荐方式并不能涵盖所有的病例, 针对每位患者, 是否需要介入诊断技术、需要何种技术或联合哪几种检查方式最为适合, 需要临床医生综合考虑每个患者病变情况及相应检查风险。PPL的最佳诊断模式还在不断探索中, 未来有望出现更多的创新!
